# Low CCL2 and CXCL8 Production and High Prevalence of Allergies in Children with Microcephaly Due to Congenital Zika Syndrome

**DOI:** 10.3390/v15091832

**Published:** 2023-08-29

**Authors:** Wallace Pitanga Bezerra, Amanda Costa Ayres Salmeron, Anna Cláudia Calvielli Castelo Branco, Ingryd Camara Morais, Valéria Soraya de Farias Sales, Paula Renata Lima Machado, Janeusa Trindade Souto, Josélio Maria Galvão de Araújo, Paulo Marcos da Matta Guedes, Maria Notomi Sato, Manuela Sales Lima Nascimento

**Affiliations:** 1 Department of Microbiology and Parasitology, Biosciences Center, Federal University of Rio Grande do Norte, Natal 59064-741, RN, Brazil; wallaceptng@gmail.com (W.P.B.); janeusatsouto@gmail.com (J.T.S.); joseliogalvao@gmail.com (J.M.G.d.A.); paulo.guedes@ufrn.br (P.M.d.M.G.); 2 Edmond and Lily Safra International Institute of Neuroscience, Santos Dumont Institute, Macaiba 59280-000, RN, Brazil; salmeron.aca@gmail.com; 3 Laboratory of Dermatology and Immunodeficiencies, LIM-56, Department of Dermatology, School of Medicine, Institute of Tropical Medicine of São Paulo, University of São Paulo, São Paulo 05403-000, SP, Brazil; annabranco@usp.br (A.C.C.C.B.); marisato@usp.br (M.N.S.); 4 Department of Immunology, Institute of Biomedical Sciences, University of São Paulo, São Paulo 05508-000, SP, Brazil; 5 Virology Laboratory, Institute of Tropical Medicine of Rio Grande do Norte, Federal University of Rio Grande do Norte, Natal 59078-190, RN, Brazil; ingrydcm@gmail.com; 6 Department of Clinical and Toxicological Analysis, Health Sciences Center, Federal University of Rio Grande do Norte, Natal 59012-570, RN, Brazil; vsales10@gmail.com (V.S.d.F.S.); paulamachado2@gmail.com (P.R.L.M.)

**Keywords:** Congenital Zika Syndrome, microcephaly, allergy, chronic inflammation, leukocyte migration

## Abstract

Congenital Zika Syndrome (CZS) is associated with an increased risk of microcephaly in affected children. This study investigated the peripheral dysregulation of immune mediators in children with microcephaly due to CZS. Gene expression quantified by qPCR in whole blood samples showed an increase in IFNγ and IL-13 transcripts in children affected with microcephaly compared to the control group. The microcephaly group exhibited significantly decreased CCL2 and CXCL8 levels in serum, quantified by CBA assay. An allergic profile questionnaire revealed a high prevalence of allergies in the microcephaly group. In accordance, elevated serum IgE level measured by the Proquantum Immunoassay was observed in children affected with microcephaly compared to the control group. Altogether, these findings show a persistent systemic inflammation in children with microcephaly due to CZS and suggest a possible impairment in leukocyte migration caused by low production of CCL2 and CXCL8, in addition to high levels of IgE associated with high prevalence of allergies. The dysregulation of inflammatory genes and chemokines underscores the importance of understanding the immunological characteristics of CZS. Further investigation into the long-term consequences of systemic inflammation in these children is crucial for developing appropriate therapeutic strategies and tailored vaccination protocols.

## 1. Introduction

Infections during the prenatal period are a significant factor contributing to fetal mortality and morbidity. These infections, along with alcohol and drug use or genetic factors, are the leading causes of congenital malformations [1]. The Zika virus (ZIKV) has been identified as an important teratogenic factor, causing the now-called Congenital Zika Syndrome (CZS). The ZIKV epidemic between April 2015 and November 2016 significantly increased the number of children born with microcephaly, characterized by occipitofrontal circumference more than two standard deviations below the median for gestational age and sex [2].

In addition to direct consequences on the central nervous system (CNS), such as developmental delay and visual and auditory impairments, other organs and systems sequelae have been described in children with microcephaly due to congenital infections, such as calcifications in the liver, spleen, and thymus; arthrogryposis; muscular hypertonia; visual and auditory problems; cardiomegaly and diaphragmatic paralysis [3,4,5,6,7]. This occurs because disruption of homeostasis triggered by intrauterine infection affects the proper development and maturation of various fetal tissues, with consequences often defined by the timing of infection during pregnancy and the pathogen involved [8].

Recently, an inflammatory imbalance has been demonstrated in neonates with CZS utilizing umbilical cord blood samples [9]. This study described an inverse relationship between head circumference and the degree of inflammation, suggesting an inflammatory biomarker signature in neonates with microcephaly due to CZS, including Interferon (IFN)γ, IFNα2, interleukin (IL)-1a, tumor necrosis factor (TNF)-α, C-C motif chemokine ligand (CCL)3, CCL4, C-X-C motif chemokine ligand (CXCL)8, CXCL10, IL-15, IL-10, IL-6, and IL-1RA. Other studies have also reported alterations in the concentrations of hepatocyte growth factor (HGF), IL-18, CXCL10, IL-1β, IL-4, IL-10, vascular endothelial growth factor (VEGF), and IFNγ in serum or cerebrospinal fluid (CSF) [10,11], although the extent of the immune system impairment in this population and the duration of these alterations remain unclear.

The leukocyte migration is an event orchestrated by chemokines and their respective receptors. Aberrant chemokines production, which is widely observed in neonates affected by CZS [9,10,11], can disrupt cell migration and affect a series of coordinated biochemical and cellular events. Likewise, CCL2 is the main chemokine involved in the recruitment of monocytes and memory T cells; its deficiency also leads to impaired memory T cell generation. On the other hand, CXCL8 is responsible for neutrophil recruitment, and, consequently, its absence impairs neutrophil migration to the tissues [12,13].

Issues underlying aspects of the immune response can have direct consequences on the body’s ability to fight pathogens. Moreover, studies describing inflammatory changes in the peripheral blood of children with CZS have predominantly focused on neonates, and it is unclear whether this imbalance persists throughout childhood. With this in mind, our group has previously identified significant morphometric alterations in the thymus and spleen of children with microcephaly due to CZS using ultrasound [6]. Additionally, we observed leukocyte morphological changes, eosinophilia, and increased serum levels of IL-2, IL-4, IL-5, and type I and II interferons. Additionally, intrauterine infections by cytomegalovirus (CMV) or Epstein-Barr virus have been associated with an increased risk of allergies and infections by intracellular pathogens, in which the child maintains a predominant Th2 response profile for a longer duration than expected, at least until the age of two [14]. Therefore, the objective of this study is to further describe the impact of CZS on the immune system and determine whether children affected by this syndrome have any degree of allergic susceptibility. We believe that elucidating the immune system panorama in children with CZS will guide future studies and contribute to the development of appropriate medical monitoring and therapeutic strategies, aiming to improve the quality of life of this pediatric population and their families.

## 2. Materials and Methods

### 2.1. Study Subjects

**Figure 1:**
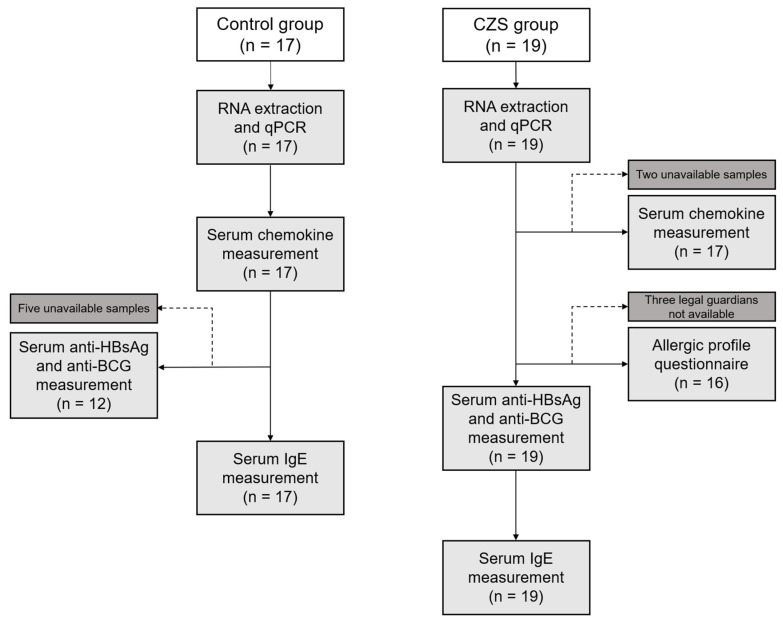
Experimental design. We recruited 19 children with microcephaly due to CZS and 17 healthy children with no neuromotor symptoms for the control group. Children from both groups had their blood drawn, and the whole blood samples were used for RNA extraction and qPCR, while the serum was used for chemokine and antibody measurements. CZS children’s legal guardians also responded to an allergic profile questionnaire. Two samples from the CZS group were unavailable for serum chemokine measurement and three of the CZS children’s legal guardians were unavailable to respond to the allergic profile questionnaire. Five samples from the control group were not available for anti-HbsAg and anti-BCG measurements.

The recruited volunteers were children between 9 to 65 months old of both sexes. The children were divided into two groups: a CZS group (n = 19), composed of children with clinical or laboratory diagnosis of congenital ZIKV infection leading to microcephaly due to CZS, according to the protocol established by the Ministry of Health or previously described [15,16] and a control group (n = 17), including healthy children with no reports of congenital infections and without any neuromotor symptoms, from the same family or neighborhood of the CZS children. Children with secondary immunodeficiencies, autoimmune conditions, symptoms of infection, or vaccinations within the past 14 days were excluded from the study. The characteristics of the individuals are shown in Table 1. All CZS participants were recruited at the Anita Garibaldi Education and Research Center for Health (CEPS) in Macaíba/RN, Brazil. We also collected urine and stool samples to test for urinary tract infections and parasitological infections, respectively; children who tested positive in any of those assays were excluded from the study. The project was approved by the Committee of Ethics in Research with Human Beings (CAAE 74871317.8.0000.5292 and 17583419.7.0000.5537). Samples were collected only after the legal guardians of the children signed an informed consent form. The legal guardians of the CZS group also completed an allergic profile questionnaire [17,18] which was corroborated by their medical records. The experimental design is shown in Figure 1.

### 2.2. Blood Sample Collection and Processing

We collected blood samples (10 mL) by venipuncture using a sterile syringe in a disposable tube with or without EDTA. The samples were processed to obtain whole blood and serum from each child for assay purposes.

### 2.3. Total RNA Extraction, Complementary DNA (cDNA) Synthesis, and qPCR

RNA was extracted from whole blood samples using the TRIzol™ reagent and the RNeasy Mini Kit (Qiagen, Hilden, Germany) according to the manufacturer’s instructions. The RNA was quantified using a Nanodrop 2000 spectrophotometer (Thermo Fisher Scientific, Waltham, MA, USA), and the samples were stored at −80 °C until further use. Complementary DNA (cDNA) was synthesized using the QuantiTect Reverse Transcription Kit (Qiagen, Hilden, Germany) following the manufacturer’s recommendations and stored at −20 °C for subsequent use in real-time PCR reactions. The real-time PCR amplification reaction was conducted in the Applied Biosystems 7500 Real-Time PCR Systems (Thermo Fisher Scientific, Waltham, MA, USA), with the Applied Biosystems SYBR Green PCR Master Mix (Thermo Fisher Scientific, Waltham, MA, USA). We used 240 ng of cDNA and 1 µM of primers for the following markers: IFNγ, IFNλ, IL-13, matrix metallopeptidase (MMP)9, retinoic acid-related orphan receptor (ROR)γt, AXL, CD209, transforming growth factor (TGF)-β, FoxP3, IL-10, and Interferon-stimulated gene (ISG)15 (Table 2). The mean of the constitutively expressed genes β-Actin and glyceraldehyde 3-phosphate dehydrogenase (GAPDH) was used for normalization. Normalized expression was calculated as previously described using the 2^−ΔCt^ formula [19], and reaction specificity was evaluated by the dissociation curve.

### 2.4. Cytometric Bead Array (CBA) for Quantification of Serum Chemokines

The chemokines CCL2, CXCL8, CXCL9, and CXCL10 were quantified in serum using the CBA method with the commercial CBA Human Chemokine Kit (BD Pharmingen, San Diego, CA, USA), following the manufacturer’s instructions. Analysis was performed on a flow cytometer (LSR Fortessa–BD Biosciences, San Diego, CA, USA), and data analysis was conducted using the FCAP Array 3.0 software (BD Biosciences, San Diego, CA, USA). The detection limits were 2.7 pg/mL (CCL2), 0.2 pg/mL (CXCL8), 2.5 pg/mL (CXCL9), and 2.8 pg/mL (CXCL10).

### 2.5. Serum IgE Measurement

Serum IgE measurement was performed using the Human IgE ProQuantum Immunoassay Kit (Invitrogen, Waltham, MA, USA), which employs a molecular method for measurement. The reactions were performed according to the manufacturer’s instructions. The measurements were performed following the manufacturer’s instruction on the 7500 Fast Real-time qPCR system (Applied Biosystems), and analysis was conducted using the ProQuantum™ on line 1.1 software (Thermo Scientific, Waltham, MA, USA). The detection limit was 0.038 pg/mL.

### 2.6. Serum Anti-HBsAg and Anti-BCG Measurements

Serum anti-HBsAg and anti-BCG measurements were performed using the Human anti-HBsAg IgG ELISA Kit (Alpha Diagnostic International, San Antonio, TX, USA) and Human anti-Tuberculosis (Bacillus Calmette-guérin/BCG; M. bovis, Tuberculosis) IgG ELISA Kit (Alpha Diagnostic International, San Antonio, TX, USA) according to the manufacturer’s instructions.

### 2.7. Statistical Analysis

Statistical analyses were performed using PRISM^®^ 6.0 software (GraphPad, Boston, MA, USA). The Agostino–Pearson and Shapiro–Wilk tests were used to assess data normality (Gaussian distribution). The Welch’s *t*-test was used to compare parametric data, while the Mann–Whitney test was utilized to compare non-parametric data. A *p*-value ≤ 0.05 was considered statistically significant.

## 3. Results

### 3.1. Children with Microcephaly Show Increased Expression of Antagonistic Cytokines IFNγ and IL-13

**Figure 2:**
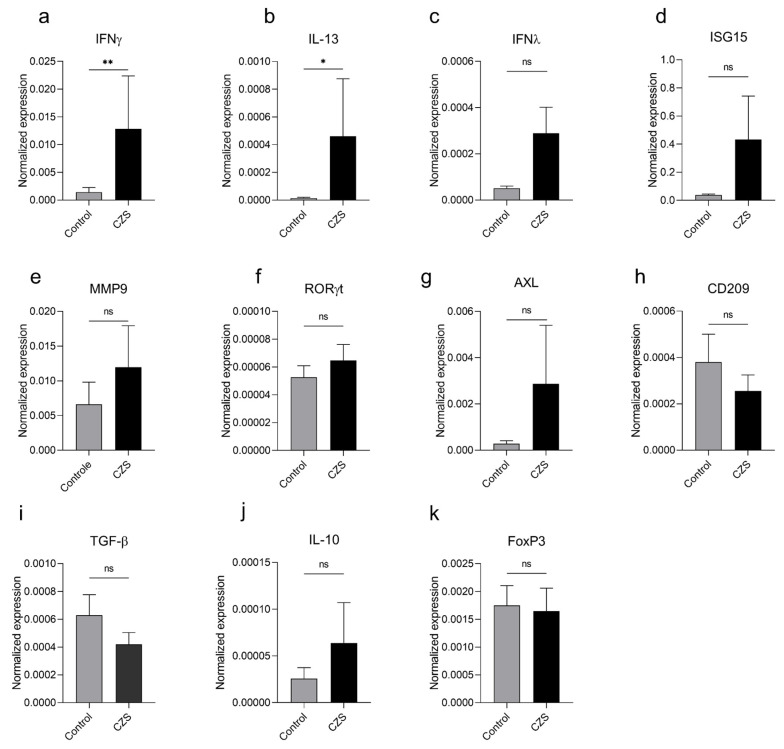
Children with microcephaly due to CZS have elevated mRNA levels of IFNγ and IL-13. qPCR on total blood samples from nineteen children with microcephaly and seventeen children from the control group was performed. The transcripts for IFNγ (**a**), IFNλ (**b**), ISG15 (**c**), IL-13 (**d**), MMP9 (**e**), RORγt (**f**), AXL (**g**), CD209 (**h**), TGF-β (**i**), FoxP3 (**j**), and IL-10 (**k**) were evaluated. The graphs represent the mean ± SEM. The Mann–Whitney test was used for all analyses. ** *p* = 0.0029, * *p* = 0.0188, ns = not significant.

Considering the previously described elevation of IL-2, IL-4, IL-5, and IFNγ in the serum of children with CZS, accompanied by peripheral blood eosinophilia [6], we aimed to evaluate the expression of other mediators that could be related to this inflammatory imbalance. We performed qPCR using total blood samples from individuals in both study groups, assessing the expression of type II and III interferons, ISG15 (antiviral and inflammatory response), IL-13 and MMP9 (Th2 profile and eosinophilic activity marker), RORγt (Th17 profile), AXL and CD209 (ZIKV entry receptors), and TGF-β, FoxP3, and IL-10 (anti-inflammatory response) (Figure 2). We observed an increase in the expression of IFNγ and IL-13 transcripts in the group of children with microcephaly compared to the control group (Figure 2a,b), while no differences in the expression of IFNλ, ISG15, MMP9, RORγt, AXL, CD209, TGF-β, FoxP3, and IL-10 were observed between the groups (Figure 2c–k).

### 3.2. Children with Microcephaly Show Marked Decreased Serum Levels of CCL2 and CXCL8

**Figure 3:**
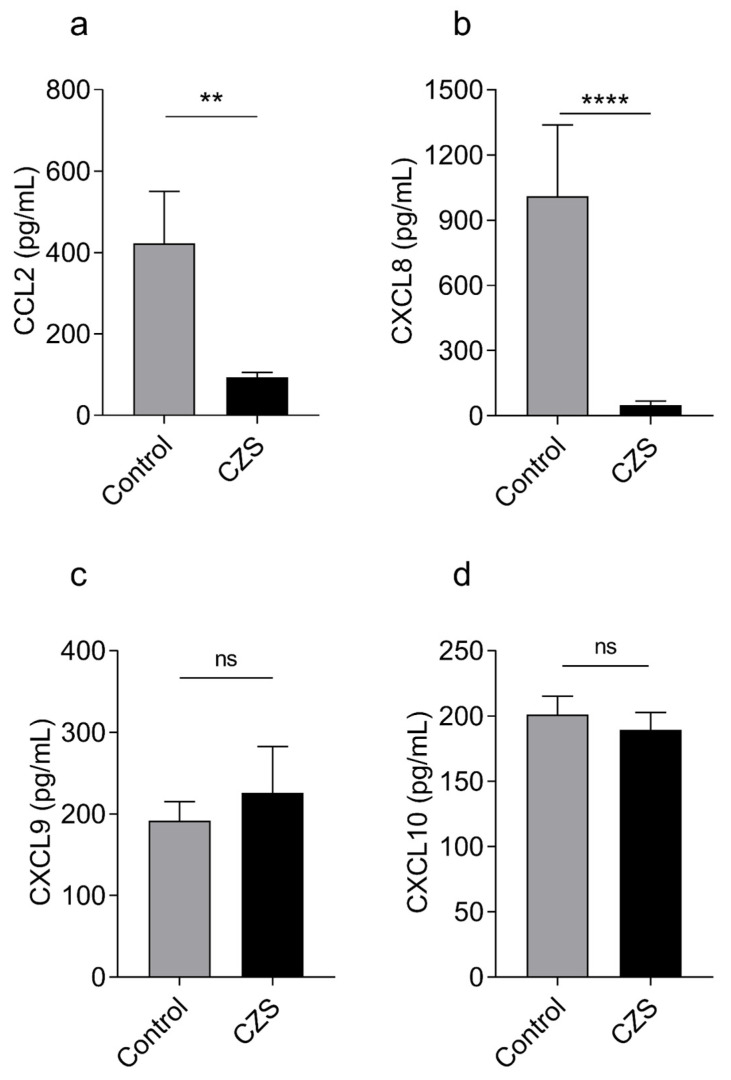
Children with microcephaly due to CZS have decreased serum levels of CCL2 and nearly absence of CXCL8. The concentration of the chemokines CXCL10 (**a**), CCL2 (**b**), CXCL9 (**c**), and CXCL8 (**d**) was measured by using CBA from the serum of sixteen children with microcephaly and seventeen children from the control group. The graphs represent the mean ± SEM. The Mann–Whitney test was used for all analyses. ** *p* = 0.0447, **** *p* = 0.0003, ns = not significant.

To better characterize the inflammatory imbalance and understand if leukocyte trafficking regulation could also be altered in patients with CZS, we measured the serum levels of the chemokines CCL2, CXCL8, CXCL9, and CXCL10, which are important for the migration of various cell types, including monocytes, macrophages, T cells, and neutrophils. Additionally, CXCL9 and CXCL10, which are inducible by IFNγ, can also modulate the phenotype of T cells by inducing the expression of the transcription factors T-box transcription factor (T-bet) and RORγt, characteristic of Th1 and Th17 profiles, respectively [20]. Children with CZS showed significantly decreased CCL2 and CXCL8 levels in serum (Figure 3a,b), while CXCL9 and CXCL10, despite the elevated levels of IFNγ, were found in similar quantities to those of the control group (Figure 3c,d).

### 3.3. Children with CZS Have an Increased Frequency of Allergies and Elevated Levels of Serum IgE

**Figure 4:**
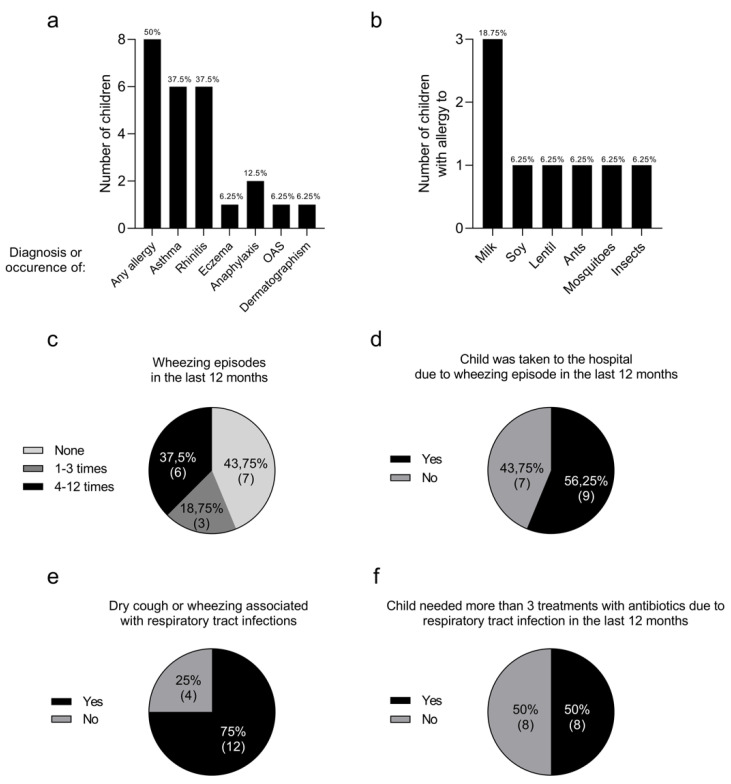
Children with microcephaly have a high prevalence of allergic conditions. Sixteen legal guardians of children with CZS answered an allergic profile questionnaire. The prevalence of aller–gic conditions (**a**), the cause of allergies (**b**), the number of wheezing episodes in the last 12 months (**c**), the need for medical attention during the episodes (**d**), the occurrence of wheezing or dry cough during respiratory infections (**e**), and whether the child received more than 3 antibiotic treatments for respiratory infections in the last 12 months (**f**) were assessed. OAS—oral allergy syndrome.

Considering that children affected with microcephaly present an increase in serum levels of Th2 cytokines such as IL-13, IL-4, and IL-5 [6], which are associated with allergic conditions such as asthma, atopic dermatitis, and food allergies, we decided to administer an allergy profile questionnaire answered by the legal guardian of the patient to evaluate this condition. Among sixteen children affected with microcephaly, half of them already had a positive diagnosis for any allergy, six (37.5%) were already diagnosed with asthma, six (37.5%) with rhinitis, one (6.25%) with previous history of eczema, two (12.5%) with previous episodes of anaphylaxis requiring medical attention, one (6.25%) with oral allergy syndrome (OAS), and one (6.25%) with a previous episode of dermographism (Figure 4a). The anaphylactic episode was caused by a medication allergy in one patient and by a food allergy in the other.

Among allergens, three children had allergies to cow’s milk, one to soy, one to lentils, one to ants, one to mosquitoes, and one to insects (Figure 4b). Additionally, out of these sixteen children, six (37.5%) had at least four wheezing episodes in the past 12 months, and three (18.75) had one to three episodes in the past 12 months (Figure 4c), with nine of them (56.25%) requiring medical attention during the episodes (Figure 4d). Twelve children (75%) had dry cough or wheezing associated with respiratory infections (Figure 4e) and eight (50%) received more than three antibiotic treatments due to respiratory infections in the past 12 months (Figure 4f).

**Figure 5:**
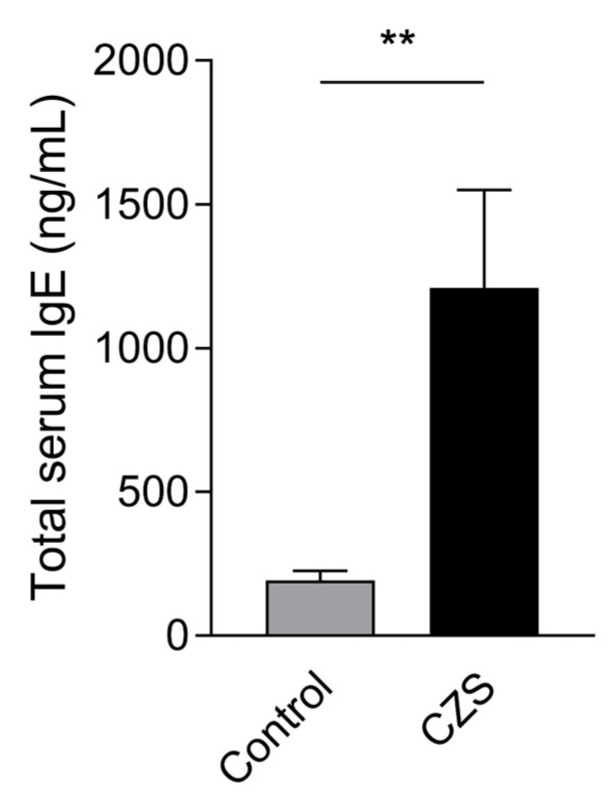
Children with microcephaly have high serum IgE levels when compared to the control group. We performed the Proquantum Immunoassay method to measure IgE levels from the serum of twenty children with microcephaly and sixteen children from the control group. The graphs represent the mean ± SEM. The Welch’s *t*-test was used for the analysis. ** *p* = 0.0079.

We then proceeded to measure serum IgE levels using the high-sensitivity Proquantum Immunoassay method. We observed an increase in serum IgE levels in the microcephalic patients compared to the control group (Figure 5), which is consistent with the questionnaire results and corroborates with the elevated levels of IL-4 in children affected with microcephaly previously described by our group [6].

## 4. Discussion

The Zika virus (ZIKV) epidemic during 2015 and 2016 was associated with an increase in cases of congenital malformations and a causal relationship was quickly established [21]. Given the central nervous system (CNS) alterations as the main symptom of CZS, efforts were focused on describing the immunopathological mechanisms of the CNS and the inflammatory response that occurs in situ in response to this infection [22,23]. However, the tropism of ZIKV is not limited to neural progenitors and in vitro studies have already demonstrated the ability of this virus to infect cells of the male and female reproductive tract, placental macrophages (Hofbauer cells), trophoblasts derived from the placenta and fibroblasts derived from skin or uterus [23,24,25].

**Figure A1:**
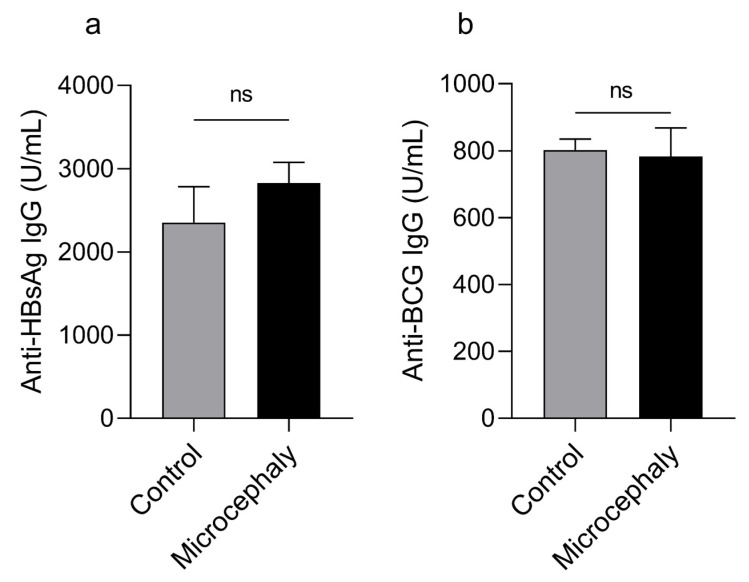
Children with microcephaly due to CZS have a normal memory humoral response to hepatitis B and BCG vaccines. IgG levels produced in response to the hepatitis B vaccine (**a**) and the BCG vaccine (**b**) were measured by ELISA. Serum from nineteen children affected with microcephaly and twelve children from the control group was analyzed, and absorbance was measured at 450 nm. The graphs represent the mean ± SEM. The Mann–Whitney test was used for all analyses. ns = not significant.

Considering the various types of cells that can be infected by ZIKV and the wide range of symptoms that patients may present, such as arthrogryposis and talipes equinovarus (clubfoot) [22], thymic alterations, and splenic calcifications [6], we hypothesize that the immune system could also be affected, and the consequences would extend beyond the neonatal period. In this study, we demonstrate that children with microcephaly due to CZS present dysregulation of antagonistic inflammatory mediators, characterized by increased mRNA expression of IFNγ and IL-13. Additionally, serum levels of CCL2 and CXCL8 are drastically decreased. Through an allergic profile questionnaire, we described a high prevalence of allergies in the microcephalic group, with a corresponding increase in serum IgE levels. Interestingly, this imbalance does not seem to have a preference for Th1 or Th2 profiles, considering the increase in IFNγ (Th1), IL-4 and IL-5 [6], and IL-13 (Th2). Despite the observed imbalance, the children demonstrated the ability to produce adequate levels of IgG antibodies in response to hepatitis B and BCG (Bacillus Calmette–Guérin) vaccines (Figure A1). We were also unable to detect the presence of ZIKV or other infections in the serum of these children that could explain the findings [6].

Our findings support previous reports of inflammatory imbalance observed in neonates with CZS [9,10,11,26], and we are the first group to demonstrate the persistence of this inflammation beyond the neonatal period [6]. These reports have shown increased levels of IL-18, IFNα, CXCL9, and CXCL10 in the serum [11] or cerebrospinal fluid [26] of neonates with CZS, and suggest a potential role of these markers in neural progenitor apoptosis during fetal development. It has also been shown that IFN-γ, IFNα2, IL-1a, TNF-α, CCL3, CCL4, CXCL8, CXCL10, IL-15, IL-10, IL-6, and IL-1RA are increased in umbilical cord blood samples from neonates born with CZS [9]. Among these markers, only IFNγ and IFNα remain elevated in children with CZS [6], while IL-6 and TNF-α levels show no difference compared to the control group. Another study reported a predominance of Th2 profile cytokines, including IL-4, IL-33, IL-37, IL-10, and TGF-β1, in the nervous tissue from fatal cases of CZS. However, in contrast with our work, IL-13 production was not increased [27]. Interestingly, IL-4 was found to be elevated in cerebrospinal fluid samples of neonates [10], while there was no increase in its production in peripheral blood or umbilical cord blood [9,11].

Among the chemokines described as elevated in neonates [9,11], CXCL8, CXCL9, and CXCL10 do not seem to persist throughout childhood in children with CZS; in contrast, we observed decreased levels of CCL2 and strikingly low levels of CXCL8 in the group of children with CZS compared to the control group. CCL2, also known as monocyte chemoattractant protein-1 (MCP-1), is a chemokine mainly involved in the recruitment of monocytes and memory T cells [28]. Its deficiency has been associated with a decrease in the generation of memory T cells and problems in their migration [29] since these cells are known to express CCR2, the receptor for CCL2. Low levels of this chemokine may, therefore, justify the weak reactor profile in tuberculin skin testing observed in children with CZS previously described [6]. Although produced by various cell types, activated monocytes/macrophages (M1) are an important source of CCL2 and CXCL8 [28,30], and macrophages derived from CCL2 knock-out (KO) mice have shown a tendency to differentiate into the M2 profile [31]. Additionally, IL-4 and IL-13, which are increased in these patients, are cytokines that polarize macrophages towards the M2 profile [30,32], and as a consequence, should induce lower amounts of CCL2 and CXCL8.

Neutrophil migration to tissues is mainly mediated by CXCL8, and its production by fibroblasts can be inhibited by type I IFNs [33], which are elevated in children with CZS [6]. Our group has already described an increase in hypersegmented and vacuolated neutrophils in the peripheral blood of children with CZS [6], which are characteristic of inflammatory conditions in which these cells persist in circulation. The low levels of CXCL8 found in this study, associated with the observed inflammatory imbalance, may, therefore, indicate that circulating neutrophils are persisting in the circulation due to low chemotaxis to tissues.

Finally, due to the increase in IL-4 and IL-5 [6], and IL-13 described in microcephalic patients and after applying the questionnaires, we observed a high prevalence of allergies among children with CZS, with frequent wheezing episodes requiring medical attention and recurrent respiratory infections requiring the use of antibiotics. Therefore, we turned to humoral immunity to assess the influence of high levels of IFNγ, IL-4, and IL-13 on IgE antibody production. IL-4 and IL-13 are cytokines related to type 2 immunity, which mediates the immune response against helminths but is also intrinsically related to allergies. IL-4 is a cytokine widely produced by Th2 and T follicular cells (T_FH_) and induces the production of IgG1 and IgE antibodies. IgE antibodies bind to their high-affinity receptor (FcεRI), which is expressed on mast cells and basophils, and upon cross-linking, induce degranulation of these cells and the release of inflammatory mediators that cause allergy symptoms [34]. IL-13, on the other hand, tends to be more prevalent in sites of active allergy, and its production induces tissue alterations such as hyperplasia of goblet cells, mucus secretion, and fibrosis [34]. Furthermore, it has been previously shown in mice that IL-13 produced by Tfh cells can qualitatively influence IgE antibodies, and in its presence, high-affinity IgE is produced [35,36]. This high-affinity IgE is better able to bind to its antigen in lower concentrations and has a better ability to induce mast cell degranulation upon allergen cross-linking [36]. Here, we did observe a difference in total IgE production between groups, marked by increased IgE levels in children with CZS, which might have increased affinity and better ability to induce allergy symptoms.

## 5. Conclusions

The findings described in this study support the hypothesis that the damage caused by congenital Zika virus infection can extend beyond the central nervous system (CNS). We demonstrated that even in the absence of symptomatic infections or asymptomatic urinary or parasitic infections, children with CZS have low CCL2 and CXCL8 production and increased serum levels of IFNγ and IL-13. Therefore, due to an inflammatory imbalance, these children may experience impairment in the migratory function of their leukocytes, particularly T cells, monocytes, and neutrophils, and functional assays may help further characterize the function of these cells in this context. Moreover, they present high levels of serum IgE and a high prevalence of allergies.

Together with previous findings [6], the alterations described here shed light on new aspects of this syndrome. This knowledge may help direct future studies and tailored therapeutic and prophylactics approaches, such as additional vaccine booster shots, especially for those vaccines that already require boosters; regular TB screening; prophylactic antibiotic therapy for those children with recurrent infections; regular visits to allergists and others. As the population of children with microcephaly due to CZS is heterogeneous, personalized evaluations are required for each patient.

## Figures and Tables

**Table 1:** Characteristics of the recruited individuals.

Patients	Sample Size	Mean Age ^#^ (±SD)	Gender
Control group	17	41.6 (±23.22)	53% Female 47% Male
CZS group	19	49.5 (±11.33)	47% Female 53% Male

CZS: Congenital Zika Syndrome; SD: standard deviation; ^#^ in months.

**Table 2:** Primers sequence used for qPCR.

Primer	Direct (5′-3′)	Reverse (5′-3′)
β-actin	GAGAGGCATCCTCACCCTGAAGTA	CACACGCAGCTCATTGTAGAAGGT
GAPDH	GAAGGTGAAGGTCGGAGT	GAAGATGGTGATGGGATTTC
IFNγ	TGTCGCCAGCAGCTAAAACA	TGCAGGCAGGACAACCATTA
IFNλ	CGCCTTGGAAGAGTCACTCA	GAAGCCTCAGGTCCCAATTC
IL-13	GCAATGGCAGCATGGTATGG	CTGCACAGTACATGCCAGCT
MMP9	AAGGATGGGAAGTACTGGCG	GCTCCTCAAAGACCGAGTCC
RORγt	TGAGAAGGACAGGGAGCCAA	CCACAGATTTTGCAAGGGATCA
AXL	CTGGGGAAGACTCTGGGAGA	CATCGTCTTCACAGCCACCT
CD209	TGCTGAGGAGCAGAACTTCC	TACTGCTTGAAGCTGGGCAA
TGFβ	GGAAATTGAGGGCTTTCGCC	AGTGAACCCGTTGATGTCCA
FoxP3	CAGCACATTCCCAGAGTTCCTC	GCGTGTGAACCAGTGGTAGATC
IL-10	TAGAGTCGCCACCCTGATGT	ACATCAAGGCGCATGTGAAC
ISG15	TGGCGGGCAACGAATT	GGGTGATCTGCGCCTTCA

## Data Availability

No new data were created or analyzed in this study. Data sharing is not applicable to this article.
